# Association between objective sleep structure and suicidal ideation in patients with depression: a study based on polysomnographic regression and cluster analysis

**DOI:** 10.3389/fpsyt.2026.1807042

**Published:** 2026-06-29

**Authors:** Renyun Zhang, Limin Yang, Xiaohua Yang, Xuefei Wu, Chao Han

**Affiliations:** 1 Shandong Mental Health Center, Shandong University, Jinan, Shandong, China; 2 Shandong Provincial Key Medical and Health Discipline of Gerontology (Shandong Mental Health Center), Jinan, Shandong, China

**Keywords:** binary logistic regression, depression, K-means cluster analysis, polysomnography, suicidal ideation

## Abstract

**Background:**

Depression is a common mental disorder, and current suicidal ideation (SI) is a clinically important symptom. Sleep disturbance is common in depression, but the relationship between objective sleep architecture and current SI remains unclear. This study examined associations between objective sleep structure and current SI and explored whether sleep-clinical clustering could support exploratory SI stratification.

**Methods:**

287 patients meeting DSM-5 criteria for depressive disorder underwent clinical assessment and overnight polysomnography (PSG). Current SI was assessed using item 3 of the HAMD-17. Binary logistic regression evaluated factors associated with current SI. K-Means clustering (K = 3) was performed using standardized HAMD score, PSQI score, AHI, total awakenings, N3%, R%, and sleep efficiency. Quantitative cluster validation, sensitivity analyses excluding HAMD from clustering, and ROC-based performance metrics were additionally conducted.

**Results:**

Current SI was present in 50.52% (145/287) of patients. In the individual-variable regression model, only HAMD total score was independently associated with current SI (OR = 1.683, p < 0.001). Cluster analysis identified three subgroups with distinct current SI rates: Cluster 1 (deep-sleep dominant, 37.04%), Cluster 2 (high-arousal-apnea, 70.37%), and Cluster 3 (low-arousal-subjective insomnia, 60.80%). The K = 3 solution showed WSS = 1450.71, mean silhouette = 0.204, and Davies-Bouldin index = 1.679. In the demographic-adjusted model, membership in Clusters 2 + 3 was associated with current SI (OR = 3.152, 95% CI: 1.883-5.274, p < 0.001; AUC = 0.700, 95% CI: 0.639-0.760). When HAMD was excluded from clustering, the association remained (OR = 2.669, 95% CI: 1.598-4.458, p < 0.001), whereas additional adjustment for HAMD total score attenuated the primary cluster association (OR = 0.842, 95% CI: 0.411-1.725, p = 0.639).

**Conclusions:**

Depression severity was the primary factor associated with current SI. PSG-derived sleep-clinical clusters may help characterize heterogeneous presentations of current SI, but their incremental value beyond depressive severity should be interpreted as exploratory and validated in larger longitudinal samples.

## 1 Introduction

Depression is a common mental disorder posing a severe threat to global public health. According to World Health Organization data, suicidal ideation among depressed patients is more frequent than in the general population, and current suicidal ideation is an important clinical warning sign for suicidal behavior (1). Previous studies have confirmed close associations between clinical factors such as depression severity, past suicide history, and suicidal ideation (2–4). However, understanding of the mechanisms involving its core comorbid symptom—sleep disturbance—remains incomplete (5).

Approximately 80% of depressed patients experience sleep disturbances (6–8). With the application of polysomnography (PSG) technology, researchers can precisely quantify sleep structure (e.g., sleep staging, microarousal index). However, existing research has largely focused on subjective sleep quality (e.g., PSQI), and conclusions regarding whether objective PSG metrics remain associated with current suicidal ideation after controlling for depression severity remain controversial (9, 10). Furthermore, single sleep metrics may fail to comprehensively reflect the complex pathological features of sleep disturbance in depression. Therefore, combining objective PSG parameters with clinical characteristics may provide a more nuanced description of sleep-related heterogeneity among patients with current suicidal ideation.

This study aims to progressively explore the relationship between sleep and current suicidal ideation using large-sample clinical data and objective PSG metrics. Specific objectives include: 1) examining the associations of clinical characteristics and individual PSG metrics with current suicidal ideation; 2) evaluating whether objective sleep metrics remain associated with current suicidal ideation after controlling for depressive symptoms; 3) identifying sleep-clinical subgroups through cluster analysis and examining their exploratory utility for stratifying current suicidal ideation.

## 2 Methods

### 2.1 Participants

**Figure 1:**
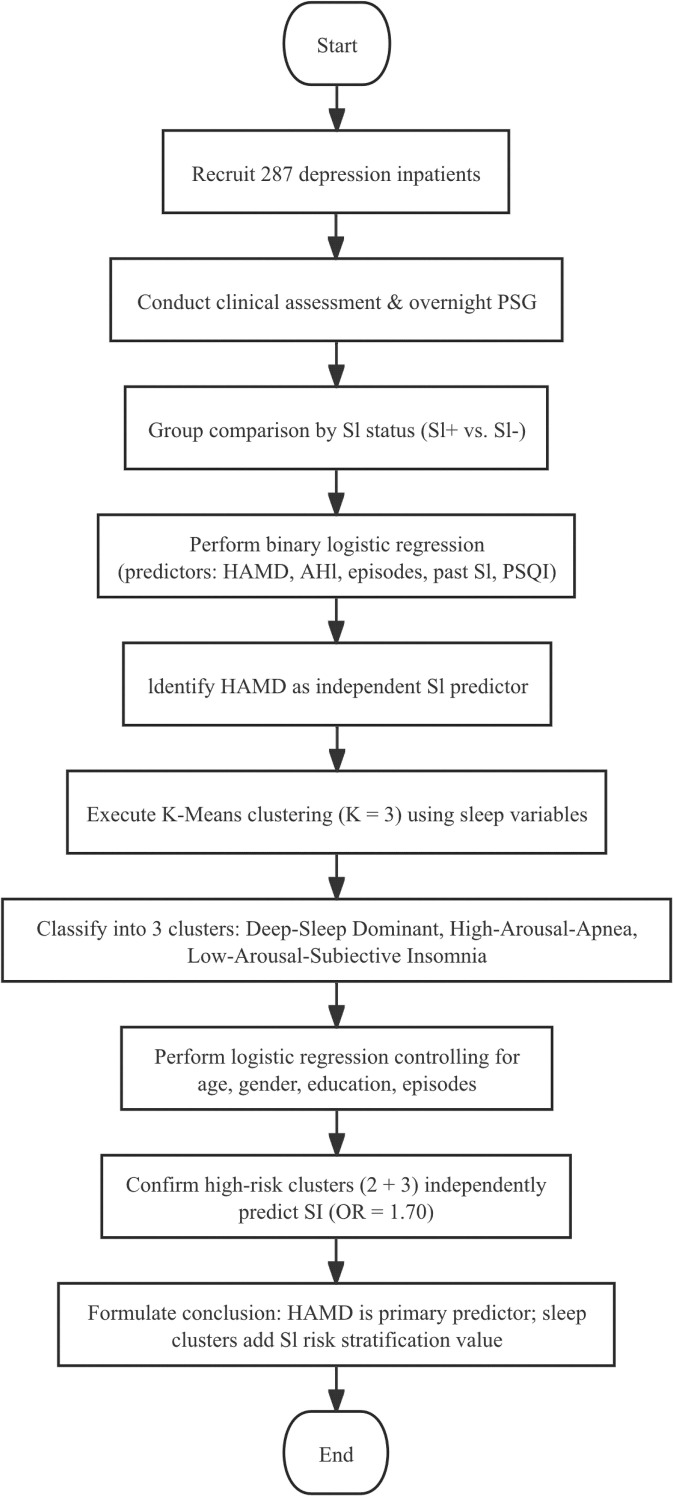
Flowchart of the study design and analytic worklow. A total of 287 inpatients with depression underwent clinicalassessment and overmight polysomnography. Paients were grouped according to current suicidal ideation (Sl) status, andlogistic regression was used to identify factors associated with current Sl. K-means clustering was then perfomed usingsleep and clinical variables to identify three sleep-clinical clusters, followed by adjusted logistic regression and ROC analysisto evaluate associations between cluster membership and current Sl. HAMD, 17-item Hamilton Depression Rating Scale.PSQl, Pitsburgh Sleep Quality lndex; AHl, apnea-hypopnea index; PSG, polysomnography; ROc, receiver operatingcharacteristic.

The sample size was estimated based on an expected medium effect size (Cohen’s f² = 0.15), α = 0.05, and 80% statistical power using G*Power 3.1 software. The minimum required sample size was 260. Ultimately, 287 inpatients with depression treated at Shandong Mental Health Center from January 2023 to December 2024 were enrolled. All patients underwent comprehensive clinical assessment and polysomnography (PSG) monitoring. A schematic overview of the study design and analytical workflow is provided in **Figure 1**. Inclusion criteria: (1) Meeting DSM-5 diagnostic criteria for depressive disorder; (2) Age 18–65 years; (3) Hamilton Depression Rating Scale (HAMD-17) total score ≥ 17; (4) Providing written informed consent. Exclusion criteria: (1) Severe physical illness or comorbid other mental disorders; (2) Recent (past 1 month) use of medications affecting sleep (e.g., benzodiazepines); (3) Poor PSG monitoring quality (effective recording < 6 hours). This study was approved by the hospital’s Ethics Committee (Approval No.: KYSJWLL2024-1-087).

### 2.2 Study variables

Dependent variable: Current suicidal ideation, assessed based on item 3 of the HAMD-17 scale (HAMD-D3), defined as a dichotomous variable (0 = absent, 1 = present).

Clinical characteristics: Included gender, age, current episode duration, number of depressive episodes, family history, education level, past suicidal ideation history (past SI), HAMD total score (depression severity), and PSQI score (subjective sleep quality, categorized as 1 = good, 2 = fair, 3 = poor, 4 = very poor). Scale assessments were conducted by two attending psychiatrists who received consistency training prior to the study (Kappa > 0.80). Unified instructions were used, and each patient’s assessment was completed by the same rater on the same day to control for inter-rater and timing biases.

Objective sleep monitoring parameters: Obtained via overnight PSG, including time in bed, total sleep time, sleep efficiency, sleep latency, percentages of sleep stages (N1%, N2%, N3%, R%), apnea-hypopnea index (AHI), total number of awakenings, and microarousal index.

### 2.3 Statistical analysis

All data analyses were performed using SPSS 26.0 and specialized statistical software, with the significance level set at p < 0.05. Additional cluster validation, sensitivity analyses, and ROC-based performance calculations were conducted using the uploaded anonymized dataset to ensure numerical consistency with the revised analyses.

Descriptive statistics and group difference tests: Categorical variables were compared using the chi-square test; continuous variables (mostly non-normally distributed) were compared between groups with and without suicidal ideation using the Mann-Whitney U test or independent samples t-test.

Correlation and regression analysis: Spearman/Pearson correlation analysis was used to explore correlations among variables. Binary logistic regression models were constructed, incorporating significantly correlated clinical and sleep variables to evaluate their independent associations with current suicidal ideation.

Cluster analysis: K-Means cluster analysis (K = 3) was performed on standardized key sleep and clinical indicators (HAMD, PSQI, AHI, total number of awakenings, N3%, R%, and sleep efficiency) to identify patient subgroups. The number of clusters was evaluated for K = 2–4 using within-cluster sum of squares (WSS; elbow method), mean silhouette coefficient, Davies-Bouldin index, cluster size, and clinical interpretability. Because HAMD total score includes HAMD item 3, which was used to define current SI, we additionally performed sensitivity analyses by rerunning the clustering without HAMD and by adding HAMD total score to the cluster-SI regression model. Predictive/classification performance of the demographic-adjusted cluster model was assessed using ROC/AUC, sensitivity, specificity, positive predictive value, negative predictive value, accuracy, Brier score, calibration intercept/slope, and the Hosmer-Lemeshow test.

## 3 Results

### 3.1 Sample characteristics

This study included a total of 287 patients with depression. Their demographic, clinical, and polysomnographic baseline characteristics are presented in **Table 1**. The mean age of patients was 38.46 ± 13.52 years, with females accounting for 59.23%. Based on the HAMD-D3 item assessment, 145 patients (50.52%) presented with suicidal ideation.

**Table 1:** Demographic, clinical, and polysomnographic characteristics of patients with depression (n = 287).

Variable	Category/statistic	Frequency (n)	Percentage (%)/statistic
Demographics			
Age (years)	Mean ± SD	287	38.46 ± 13.52
Gender	Female	170	59.23
	Male	117	40.77
Education Level	1 (Primary school or below)	52	18.12
	2 (Junior high school)	70	24.39
	3 (High school/Technical secondary)	109	37.98
	4 (College or above)	56	19.51
Clinical Characteristics			
HAMD-17 total score	Mean ± SD	287	28.42 ± 5.60
Current episode duration (months)	Median (IQR)	287	2.50 (4.00)
Number of episodes	0	2	0.70
	1	107	37.28
	2	97	33.80
	3	45	15.68
	4	33	11.50
	5	3	1.05
Family history of mental illness	No	245	85.37
	Yes	42	14.63
History of past suicidal ideation (Past SI)	No	170	59.23
	Yes	117	40.77
PSQI score (subjective sleep quality)	2	5	1.74
	3	137	47.74
	4	145	50.52
Polysomnography (PSG) Parameters			
Total sleep time (min)	Mean ± SD	287	476.32 ± 88.46
Sleep efficiency	Mean ± SD	287	0.86 ± 0.12
N1%	Mean ± SD	287	0.13 ± 0.09
N2%	Mean ± SD	287	0.60 ± 0.16
N3%	Mean ± SD	287	0.15 ± 0.14
R%	Mean ± SD	287	0.12 ± 0.07
Apnea-Hypopnea Index (AHI, events/hour)	Median (IQR)	287	2.00 (6.90)
Total number of awakenings	Mean ± SD	287	71.18 ± 69.27
Microarousal index	Mean ± SD	287	9.11 ± 9.11

HAMD, Hamilton Depression Rating Scale; PSQI, Pittsburgh Sleep Quality Index; AHI, Apnea-Hypopnea Index; N1%, N2%, and N3%, proportion of stage N1, N2, and N3 non-rapid eye movement sleep; R%, proportion of rapid eye movement sleep; IQR, interquartile range; SD, standard deviation.

Clinical characteristics indicated that the overall depressive symptoms were at a moderate to severe level (HAMD-17 total score: 28.42 ± 5.60). The current episode duration showed a right-skewed distribution (median = 2.50 months, IQR = 4.00 months). Regarding illness history, the majority of patients (71.08%) had experienced 1–2 episodes, most had no family history of mental illness (85.37%), and 40.77% of patients reported a history of past suicidal ideation. Subjective sleep quality assessment (PSQI) revealed that the vast majority of patients (98.26%) had sleep quality ranging from fair to very poor.

Objective parameters from overnight polysomnography showed that the patients’ mean total sleep time was 476.32 ± 88.46 minutes, and the mean sleep efficiency was 0.86 ± 0.12. The median Apnea-Hypopnea Index (AHI) was 2.00 events/hour. Sleep structure analysis indicated that the percentages of N1, N2, N3, and REM sleep stages were 13 ± 9%, 60 ± 16%, 15 ± 14%, and 12 ± 7%, respectively (detailed descriptive statistics for all PSG parameters are available in **Supplementary Materials**).

### 3.2 Group differences based on suicidal ideation status

The suicidal ideation positive group had a significantly higher HAMD total score than the negative group (31.77 ± 5.28 vs 24.99 ± 3.42, t = -12.878, p < 0.001), and a higher AHI (7.32 ± 12.19 vs 4.72 ± 9.58, t = -2.010, p = 0.045) (**Table 2**). Among categorical variables, the prevalence of past suicidal ideation history (χ² = 23.995, p < 0.001), the proportion of patients with ≥ 3 episodes (χ² = 21.125, p = 0.001), and the proportion with poorer PSQI scores (score of 4) (χ² = 11.291, p = 0.004) were significantly higher in the positive group. There were no significant differences between the two groups in age, gender, or other categorical variables listed (p > 0.05; complete results for all continuous and categorical variables are provided in **Supplementary Materials**).

**Table 2:** Comparison of characteristics between depressed patients with and without suicidal ideation.

Variable	No suicidal ideation (n = 142)	Suicidal ideation (n = 145)	Statistic	p-value
Continuous Variables [Mean ± SD]				
HAMD total score	24.99 ± 3.42	31.77 ± 5.28	t = -12.878	< 0.001
AHI (events/hour)	4.72 ± 9.58	7.32 ± 12.19	t = -2.010	0.045
Age (years)	39.75 ± 12.90	37.19 ± 14.03	t = 1.613	0.108
Categorical Variables [n (%)]				
Number of episodes (≥ 3)	24 (16.90)	57 (39.31)	χ² = 21.125	0.001
History of past suicidal ideation (Yes)	37 (26.06)	80 (55.17)	χ² = 23.995	< 0.001
PSQI score (4, Poor)	58 (40.85)	87 (60.00)	χ² = 11.291	0.004
Gender (Male)	53 (37.32)	64 (44.14)	χ² = 1.112	0.292

This table presents key variables with statistically significant differences or demographics. Complete results for all variables are available in **Supplementary Materials**. SD, standard deviation.

### 3.3 Correlation analysis

Pearson correlation analysis showed that HAMD score was significantly positively correlated with PSQI score (r = 0.353, p < 0.01) and AHI (r = 0.119, p < 0.05); it was significantly negatively correlated with the total number of awakenings (r = -0.139, p < 0.05) and the microarousal index (r = -0.144, p < 0.05). Other objective sleep structure metrics (e.g., total sleep time, sleep efficiency, percentages of sleep stages) showed no significant correlation with the HAMD score (p > 0.05; the full correlation matrix is provided in **Supplementary Materials**).

### 3.4 Binary logistic regression analysis

Binary logistic regression models incorporated variables that were significant in univariate analyses or of key interest: HAMD total score, AHI, number of episodes, history of past suicidal ideation, and PSQI total score. The overall model fit was significant (Likelihood Ratio χ² = 178.224, df = 5, p < 0.001; Nagelkerke R² = 0.617). Results showed that the HAMD total score was the only independent factor associated with current suicidal ideation in this model (B = 0.521, SE = 0.063, Wald χ² = 67.743, p < 0.001, OR = 1.683, 95% CI: 1.487 – 1.906). AHI, number of episodes, past suicidal ideation history, and PSQI score did not show independent statistical significance (p > 0.05) (**Table 3**).

**Table 3:** Binary logistic regression analysis of factors associated with suicidal ideation.

Predictor	B (β)	SE	Wald χ²	p-value	OR	95% CI for OR
HAMD total score	0.521	0.063	67.743	< 0.001	1.683	1.487 – 1.906
AHI	0.015	0.015	0.937	0.333	1.015	0.985 – 1.046
Number of episodes	0.154	0.205	0.565	0.452	1.167	0.780 – 1.745
History of past suicidal ideation	0.468	0.397	1.390	0.238	1.598	0.734 – 3.479
PSQI total score	-0.390	0.339	1.325	0.250	0.677	0.349 – 1.315
Constant	-13.841	1.904	52.820	< 0.001	< 0.001	–
Model Fit Indices						
-2 Log likelihood	219.611					
Model χ² (df = 5)	178.224			< 0.001		
Nagelkerke R²	0.617					

Dependent variable: presence of suicidal ideation (HAMD item 3). Variables entered were those associated with suicidal ideation in univariate analyses. OR, odds ratio; CI, confidence interval; SE, standard error.

### 3.5 Cluster analysis results and association with current suicidal ideation

K-Means clustering (K = 3) based on HAMD score, PSQI score, AHI, total awakenings, N3%, R%, and sleep efficiency divided the patients into three sleep-clinical subgroups (**Table 4**). Quantitative validation showed the following WSS, silhouette, and Davies-Bouldin values: K = 2, 1655.05, 0.199, and 1.940; K = 3, 1450.71, 0.204, and 1.679; and K = 4, 1297.09, 0.211, and 1.515. Although the Davies-Bouldin index continued to improve modestly at K = 4, the K = 4 solution introduced an additional small subgroup with limited incremental interpretability. Therefore, K = 3 was retained as a parsimonious and clinically interpretable solution. Cluster 1 (n = 135, 47.0%), termed “Deep-Sleep Dominant”, exhibited the highest proportion of deep sleep (N3% = 0.198 ± 0.148) and low AHI. Cluster 2 (n = 27, 9.4%), termed “High-Arousal-Apnea”, was characterized by high AHI (35.144 ± 14.727), low N3% (0.066 ± 0.104), and increased awakenings (113.000 ± 86.42). Cluster 3 (n = 125, 43.6%), termed “Low-Arousal-Subjective Insomnia”, presented with the lowest number of awakenings (45.86 ± 36.06), moderately low N3% and R%, and poor subjective sleep quality. The chi-square test showed a significant association between cluster grouping and current SI (χ² = 19.358, df = 2, p < 0.001): 37.04% in Cluster 1, 70.37% in Cluster 2, and 60.80% in Cluster 3. Given the small size of Cluster 2, its profile should be interpreted as exploratory.

**Table 4:** Sleep-clinical clusters identified and their association with suicidal ideation.

Item	Cluster 1 (deep-sleep dominant, n = 135)	Cluster 2 (high-arousal-apnea, n = 27)	Cluster 3 (low-arousal-subjective insomnia, n = 125)	Statistical test (p-value)
Cluster Features [Mean ± SD]				
HAMD total score	26.15 ± 3.92	31.70 ± 10.36	30.16 ± 4.71	F = 25.538 (< 0.001)
PSQI total score	2.99 ± 0.21	3.74 ± 0.53	3.98 ± 0.15	F = 579.385 (< 0.001)
AHI (events/hour)	2.97 ± 3.79	35.14 ± 14.73	3.06 ± 4.02	F = 374.570 (< 0.001)
Total number of awakenings	86.27 ± 80.06	113.00 ± 86.42	45.86 ± 36.06	F = 18.489 (< 0.001)
N3%	0.198 ± 0.148	0.066 ± 0.104	0.121 ± 0.118	F = 17.314 (< 0.001)
Suicidal Ideation [n (%)]				
Presence of suicidal ideation	50 (37.04%)	19 (70.37%)	76 (60.80%)	χ² = 19.358 (< 0.001)

Cluster analysis was performed using HAMD, PSQI, AHI, total awakenings, N3%, R%, and sleep efficiency. Detailed ANOVA results for R% and sleep efficiency are provided in **Supplementary Materials**.

### 3.6 Clinical characteristics across cluster subgroups

Clinical background factors showed a heterogeneous distribution across cluster subgroups (**Table 5**). Age differed significantly among the three subgroups (F = 12.586, p < 0.001), with Cluster 2 being significantly older than both Cluster 1 and Cluster 3. Analysis of categorical variables showed significant differences in gender distribution (χ² = 14.536, p = 0.001), number of episodes (≥ 3 episodes; χ² = 19.483, p = 0.035), and education level (χ² = 19.909, p = 0.003). Family history and past suicidal ideation history did not show significant differences in distribution across subgroups (p > 0.05). The complete table comparing all clinical characteristics across clusters is available in **Supplementary Materials**.

**Table 5:** Distribution of clinical background characteristics across cluster subgroups.

Variable	Cluster 1 (n = 135)	Cluster 2 (n = 27)	Cluster 3 (n = 125)	Statistical test (p-value)
Continuous Variables [Mean ± SD]				
Age (years)	35.97 ± 12.35	49.70 ± 11.98	38.71 ± 13.88	F = 12.586 (< 0.001)
Current episode duration (months)	3.55 ± 4.06	3.29 ± 3.37	4.58 ± 4.87	F = 2.166 (0.117)
Categorical Variables [n (%)]				
Gender (Female)	81 (60.00)	7 (25.93)	82 (65.60)	χ² = 14.536 (0.001)
Number of episodes (≥ 3)	30 (22.22)	9 (33.33)	42 (33.60)	χ² = 19.483 (0.035)
Education level (Low: Level 1-2)	55 (40.74)	14 (51.85)	53 (42.40)	χ² = 19.909 (0.003)
History of past suicidal ideation (Yes)	49 (36.30)	13 (48.15)	55 (44.00)	χ² = 2.268 (0.322)

Age in Cluster 2 was significantly higher than in Clusters 1 and 3 (p < 0.001). Complete comparison of all clinical characteristics is provided in **Supplementary Materials**.

### 3.7 Cluster association, sensitivity analyses, and classification performance

Binary logistic regression was used to evaluate the association between high-risk cluster membership (Clusters 2 + 3 vs. Cluster 1) and current suicidal ideation after controlling for age, gender, number of episodes, and education level. The overall model was significant (Likelihood Ratio χ² = 39.952, df = 5, p < 0.001; Nagelkerke R² = 0.173). High-risk cluster membership was associated with current SI (B = 1.148, SE = 0.263, Wald χ² = 19.095, p < 0.001, OR = 3.152, 95% CI: 1.883 – 5.274), and number of episodes was also associated with current SI (OR = 1.546, 95% CI: 1.205 – 1.984, p < 0.001) (**Table 6**). The demographic-adjusted cluster model showed modest discrimination (AUC = 0.700, 95% CI: 0.639 – 0.760), with sensitivity = 0.462 and specificity = 0.908 at the Youden threshold. Sensitivity analysis excluding HAMD from the clustering produced a similar three-cluster pattern (n = 135/26/126) and a persistent demographic-adjusted high-risk association (OR = 2.669, 95% CI: 1.598 – 4.458, p < 0.001). However, when HAMD total score was added to the primary cluster association model, high-risk cluster membership was attenuated and no longer statistically significant (OR = 0.842, 95% CI: 0.411 – 1.725, p = 0.639), whereas HAMD remained strongly associated with current SI (OR = 1.679, 95% CI: 1.484 – 1.900, p < 0.001) (**Supplementary Materials**).

**Table 6:** Demographic-adjusted association of high-risk cluster group with current suicidal ideation.

Predictor	B (β)	SE	Wald χ²	p-value	OR	95% CI for OR
High-risk cluster subgroup	1.148	0.263	19.095	< 0.001	3.152	1.883 – 5.274
Number of episodes	0.436	0.127	11.757	< 0.001	1.546	1.205 – 1.984
Age (years)	-0.019	0.010	3.394	0.065	0.981	0.961 – 1.001
Gender (Male)	0.301	0.258	1.362	0.243	1.352	0.815 – 2.242
Education level	0.204	0.139	2.165	0.141	1.226	0.935 – 1.609
Constant	-1.381	0.706	3.824	0.051	0.251	0.063 – 1.003
Model Fit Indices						
-2 Log likelihood	357.883					
Model χ² (df = 5)	39.952			< 0.001		
Nagelkerke R²	0.173					

Dependent variable: current suicidal ideation. The cluster subgroup is a binary variable (High-risk: Clusters 2 & 3 combined; reference: Cluster 1). This model adjusts for age, gender, number of episodes, and education level; the HAMD-adjusted sensitivity model is reported in **Supplementary Materials**.

## 4 Discussion

This study, utilizing polysomnographic data from a large sample of depressed patients combined with regression and cluster analysis, aimed to clarify how objective sleep architecture and sleep-clinical profiles are associated with current suicidal ideation. The results demonstrate that depression severity, as measured by the HAMD total score, is the principal factor associated with current suicidal ideation, a finding consistent with previous research and underscoring the central role of core depressive symptoms (11). Individual PSG variables did not remain independently associated with current SI in the multivariable clinical model. Cluster analysis identified clinically interpretable sleep-clinical profiles with different current SI rates; however, the additional HAMD-adjusted sensitivity analysis indicates that the cluster-SI association is partly explained by depressive symptom severity. Thus, the cluster findings should be viewed as exploratory stratification of current SI presentation rather than evidence of causal, prognostic, or depression-severity-independent prediction.

### 4.1 Patient demographics and clinical context for current suicidal ideation assessment

The characteristics of the 287 depressed patients provide essential context for interpreting the relationship between depression, sleep, and suicidal ideation. The mean age was 38.46 ± 13.52 years, with females accounting for 59.23%, consistent with epidemiological evidence suggesting that females may face a higher risk of depression onset, potentially due to interactions among hormonal fluctuations, social stress, or genetic factors (12). The high prevalence in young and middle-aged groups may also reflect modern life stress and occupational burnout (13), highlighting the need for screening tools tailored to different demographic profiles for early identification of potential risks. Furthermore, 40.77% of patients reported a history of past suicidal ideation, a proportion significantly higher in the group currently expressing suicidal ideation, which supports the continuity and recurrence model of this symptom within the course of depression. Past history, as a strong predictor, may reflect inherent cognitive bias or neurobiological vulnerability (14–16). Systematic assessment of past history at initial diagnosis, supplemented by standardized questionnaires alongside HAMD evaluation, is therefore crucial for achieving comprehensive risk stratification. The association between frequent episodes and depression severity was also evident; although most patients had 1–2 episodes, the proportion with more than 2 episodes was higher in the suicidal ideation group, possibly due to accumulated neuroinflammation or increased treatment resistance resulting from disease chronicity. For such patients, intensified interventions combining cognitive behavioral therapy and pharmacotherapy should be prioritized to interrupt the vicious cycle and improve long-term prognosis.

### 4.2 Clinical correlates of current suicidal ideation and their implications

The regression analysis clarified the clinical correlates of current suicidal ideation within the framework of depression. HAMD total score was the only independent factor in the individual-variable regression model (B = 0.521, p < 0.001, OR = 1.683), reinforcing that overall depressive symptom severity is closely linked to current SI (17–19). In contrast, AHI, past suicidal ideation history, PSQI score, and number of episodes did not retain independent significance in that HAMD-inclusive model. Number of episodes remained associated with current SI in the demographic-adjusted cluster model, but this should be interpreted as an illness-course correlate rather than a stand-alone prognostic factor. These findings do not diminish the clinical importance of sleep disturbance; rather, they suggest that single PSG metrics provide limited additional information once overall depressive severity is considered. Clinically, the results support careful assessment of depressive severity and current SI, while sleep variables may be most informative when interpreted as part of broader clinical profiles.

### 4.3 Sleep-clinical clusters and their exploratory application in current suicidal ideation assessment

Given the limitations of individual sleep metrics, we used cluster analysis to identify multidimensional sleep-clinical patterns that might describe clinically relevant heterogeneity. This approach revealed three subgroups with different current SI rates. Cluster 1, termed “Deep-Sleep Dominant,” was characterized by the highest proportion of deep sleep (N3% = 0.198) and low AHI (2.97 ± 3.79), and exhibited the lowest current SI rate (37.04%). Cluster 2, “High-Arousal-Apnea,” presented with high AHI (35.14 ± 14.73), low N3% (0.066), and increased awakenings (113.00 ± 86.42), alongside the highest current SI rate (70.37%). Because this subgroup contained only 27 patients, mechanistic interpretation should remain cautious. Its profile is compatible with, but does not prove, possible links among sleep-disordered breathing, sleep fragmentation, nocturnal hypoxia, inflammation, and emotional regulation (20, 21). Cluster 3, “Low-Arousal-Subjective Insomnia,” was marked by fewer awakenings (45.86 ± 36.06), moderately reduced N3% and R%, and the poorest subjective sleep quality (PSQI = 3.98), with a current SI rate of 60.80%. This subjective-objective discrepancy may reflect cognitive or affective factors related to sleep perception, but this interpretation requires confirmation in future studies (22).

The clustering incorporated both PSG indices and clinical measures (HAMD and PSQI) to capture the multidimensional nature of depression-related sleep dysfunction. However, because HAMD total score includes item 3, which defined current SI, we acknowledge the potential for partial circularity. The HAMD-excluded clustering sensitivity analysis produced similar broad profiles and retained a demographic-adjusted association with current SI, suggesting that the cluster structure was not solely created by HAMD. Nevertheless, the primary cluster association was no longer significant after additional adjustment for HAMD total score. Therefore, the clusters should be interpreted as descriptive sleep-clinical phenotypes associated with current SI rather than as independent predictors beyond depressive severity.

These exploratory profiles may still have clinical relevance when used cautiously. Patients resembling Cluster 2 may warrant closer assessment for sleep-disordered breathing and sleep fragmentation, including consideration of evidence-based sleep apnea management when clinically indicated (23). Patients with low N3% may be candidates for careful evaluation of sleep continuity and slow-wave sleep disturbance, while patients resembling Cluster 3 may benefit from assessment of subjective insomnia, rumination, and sleep-state misperception, for which CBT-I may be relevant (24). At this stage, these implications should be regarded as hypotheses for future testing rather than definitive cluster-specific treatment recommendations.

### 4.4 Classification performance and translational considerations

The additional classification analysis provides a preliminary estimate of clinical utility for current SI stratification rather than prognostic evidence. The demographic-adjusted cluster model showed modest discrimination (AUC = 0.700), high specificity (0.908), and lower sensitivity (0.462), indicating that the model was better at identifying patients without current SI than detecting all patients with current SI. Calibration indices were acceptable in this dataset (Brier score = 0.218; calibration slope = 1.000; Hosmer-Lemeshow p = 0.067), but these internal metrics require external validation. When HAMD total score was added, model discrimination increased substantially (AUC = 0.908), but the cluster effect was attenuated, indicating that this improvement was mainly driven by depressive symptom severity. Therefore, cluster outputs should not be used as stand-alone predictive tools. Future longitudinal and multicenter studies should test whether these sleep-clinical profiles remain stable over time, predict subsequent suicidal behavior, or moderate response to sleep-focused interventions.

Several limitations should be considered. First, the sample was drawn from a single tertiary psychiatric center, which may limit generalizability. Second, the cross-sectional design precludes causal or prognostic inference; all findings refer to current suicidal ideation measured at the time of assessment. Third, Cluster 2 was relatively small (n = 27), so its high-arousal-apnea profile and related mechanistic explanations should be regarded as exploratory and require validation in larger samples. Fourth, inclusion of HAMD total score in the clustering variables may introduce partial circularity because HAMD item 3 defined current SI. We addressed this by conducting HAMD-excluded clustering and HAMD-adjusted sensitivity analyses, but future studies should use item-level HAMD data or independent suicidal ideation instruments to avoid part-whole overlap. Fifth, residual confounding by unmeasured factors (e.g., medication history, psychosocial stressors) cannot be excluded. Finally, current SI was defined by a single HAMD-17 item, which may not fully capture the complexity of suicidal ideation.

## 5 Conclusion

This study indicates that depression severity is the primary factor associated with current suicidal ideation among patients with depression. Individual objective PSG metrics did not show independent associations after accounting for clinical variables, whereas sleep-clinical clusters described heterogeneous profiles with different current SI rates. These cluster findings may support exploratory stratification and hypothesis generation, but their incremental value beyond depressive severity and their prognostic utility require validation in larger longitudinal cohorts using independent suicidal ideation measures.

## Data Availability

The raw data supporting the conclusions of this article will be made available by the authors, without undue reservation.

## References

[1] Sokero TP Melartin TK Rytsälä HJ Leskelä US Lestelä-Mielonen PS Isometsä ET . Suicidal ideation and attempts among psychiatric patients with major depressive disorder. J Clin Psychiatry. (2003) 64:1094–100. doi: 10.4088/jcp.v64n0916 14628986

[2] Alexopoulos GS Bruce ML Hull J Sirey JA Kakuma T . Clinical determinants of suicidal ideation and behavior in geriatric depression. Arch Gen Psychiatry. (1999) 56:1048–53. doi: 10.1001/archpsyc.56.11.1048 10565506

[3] Lage RR Rafael D Tancini MB Nardi AE Mograbi DC Cheniaux E . Suicidal ideation in bipolar disorder: The relation with clinical and sociodemographic variables. Psychiatr Q. (2022) 93:453–61. doi: 10.1007/s11126-021-09965-0 34664176

[4] Valtonen H Suominen K Mantere O Leppmki S Isomets ET . Suicidal ideation and attempts in bipolar I and II disorders. J Clin Psychiatry. (2005) 66:1456–62. doi: 10.4088/jcp.v66n1116 16420084

[5] Paulekiene G Pajarskiene M Pajediene E Radziunas A . Sleep dysfunction and grey matter volume. Curr Neurol Neurosci Rep. (2022) 22:275–83. doi: 10.1007/s11910-022-01190-x 35364772

[6] Seow L Subramanian M Abdin E Vaingankar J Chong S . Sleep disturbance among people with major depressive disorders (MDD) in Singapore. J Ment Health. (2015) 16:S259–60. doi: 10.1016/j.sleep.2015.02.1564 27935392

[7] Dealberto MJ . Sleep disorders in psychiatric diseases. Epidemiological aspects. Encephale-revue Psychiatr Clin Biol Et Therapeutique. (1992) 18:331–40. 1297583

[8] Franzen PL Buysse DJ . Sleep disturbances and depression: risk relationships for subsequent depression and therapeutic implications. Dialogues Clin Neurosci. (2008) 10:473–81. doi: 10.31887/dcns.2008.10.4/plfranzen 19170404 PMC3108260

[9] Zeoli I Lanquart JP Wacquier B Mungo A Loas G Hein M . Polysomnographic markers of suicidal ideation in untreated unipolar major depressed individuals. Int J Psychophysiol. (2021) 166:19–24. doi: 10.1016/j.ijpsycho.2021.05.001 33965422

[10] Bernert RA Luckenbaugh DA Duncan WC Iwata M Zarate CA . Sleep architecture parameters as a putative biomarker of suicidal ideation in treatment-resistant depression. J Affect Disord. (2017) 208:309–15. doi: 10.1016/j.jad.2016.08.050 27810712 PMC6502232

[11] Keilp JG Grunebaum MF Gorlyn M LeBlanc S Burke AK Galfalvy H . Suicidal ideation and the subjective aspects of depression. J Affect Disord. (2012) 140:75–81. doi: 10.1016/j.jad.2012.01.045 22406338 PMC3375058

[12] Albert KM Newhouse PA . Estrogen, stress, and depression: Cognitive and biological interactions. Annu Rev Clin Psychol. (2019) 15:399–423. doi: 10.1146/annurev-clinpsy-050718-095557 30786242 PMC9673602

[13] Maslach C Schaufeli WB Leiter MP . Job burnout. Annu Rev Psychol. (2001) 52:397–422. doi: 10.1111/1467-8721.01258 11148311

[14] Brent DA Kolko DJ Wartella ME Boylan MB Moritz G Baugher M . Adolescent psychiatric inpatients' risk of suicide attempt at 6-month follow-up. J Am Acad Child Adolesc Psychiatry. (1993) 32:95–105. doi: 10.1097/00004583-199301000-00015 8428891

[15] Kivelä LMM Antypa N Fried EI Schoevers R van Hemert AM Penninx BWJH . Suicidal ideation across depressive episodes: 9-year longitudinal cohort study. BJPsych Open. (2023) 9:e218. 37981566 10.1192/bjo.2023.608 PMC10755669

[16] Roy-Byrne PP Post RM Hambrick DD Leverich GS Rosoff AS . Suicide and course of illness in major affective disorder. J Affect Disord. (1988) 15:1–8. doi: 10.1016/0165-0327(88)90002-x 2970487

[17] Merriam EP Thase ME Haas GL Keshavan MS Sweeney JA . Prefrontal cortical dysfunction in depression determined by Wisconsin Card Sorting Test performance. Am J Psychiatry. (1999) 156:780–2. doi: 10.1176/ajp.156.5.780 10327916

[18] Kim YK Han KM . Neural substrates for late-life depression: A selective review of structural neuroimaging studies. Prog Neuro-Psychopharmacol Biol Psychiatry. (2021) 104:110010. doi: 10.1016/j.pnpbp.2020.110010 32544600

[19] Miller CH Hamilton JP Sacchet MD Gotlib IH . Meta-analysis of functional neuroimaging of major depressive disorder in youth. JAMA Psychiatry. (2015) 72:1045–53. doi: 10.1001/jamapsychiatry.2015.1376 26332700 PMC11890701

[20] Thase ME . Depression, sleep, and antidepressants. J Clin Psychiatry. (1998) 59:55–65. 9554322

[21] Yang C Zhou Y Liu H Xu P . The role of inflammation in cognitive impairment of obstructive sleep apnea syndrome. Brain Sci. (2022) 12:1303. doi: 10.3390/brainsci12101303 36291237 PMC9599901

[22] DiNapoli EA Gebara MA Kho T Butters MA Gildengers AG Albert SM . Subjective-objective sleep discrepancy in older adults with MCI and subsyndromal depression. J Geriatric Psychiatry Neurol. (2017) 30(6):316–23. doi: 10.1177/0891988717731827 28954595 PMC5916761

[23] Yu L Shu L Gao S Li L . CPAP improves sleep stability and attenuates acute nocturnal hypertension (NBPF) in OSA, with maximal benefits in severe cases. Front Neurol. (2025) 16:1587127. doi: 10.3389/fneur.2025.1587127 40692557 PMC12277164

[24] Roniger DDG Lechuga YA León EE Robles ROG Escandón ÓS Pérez GuT . Cognitive behavioral therapy for insomnia helps to reverse cognitive impairment in insomnia patients. Sleep Sci. (2022) 15:355–60. doi: 10.5935/1984-0063.20210026 35371411 PMC8906373

