# Supplementary material for: Association between objective sleep structure and suicidal ideation in patients with depression: a study based on polysomnographic regression and cluster analysis
Source: Front Psychiatry. 2026 Jun 29;17:1807042. doi: 10.3389/fpsyt.2026.1807042 (PMC13357665; doi:10.3389/fpsyt.2026.1807042)
Supplement: Supplementary file 1 [file Table1.docx]

**Supplementary Table. Quantitative Cluster Validation, Sensitivity Analyses, and Classification Performance for Current Suicidal Ideation**

| Analysis | Metric / model | Result | Interpretation |
| --- | --- | --- | --- |
| K = 2 validation | WSS; silhouette; Davies-Bouldin; cluster sizes | 1655.05; 0.199; 1.940; 141/146 | Two clusters merged clinically distinct sleep profiles. |
| K = 3 validation | WSS; silhouette; Davies-Bouldin; cluster sizes | 1450.71; 0.204; 1.679; 135/27/125 | Retained as the most parsimonious clinically interpretable solution. |
| K = 4 validation | WSS; silhouette; Davies-Bouldin; cluster sizes | 1297.09; 0.211; 1.515; 116/39/111/21 | The modest metric improvement was accompanied by an additional small subgroup. |
| Primary cluster model | ROC/AUC and classification at Youden threshold | AUC = 0.700 (95% CI: 0.639-0.760); sensitivity = 0.462; specificity = 0.908; PPV = 0.838; NPV = 0.623; accuracy = 0.683 | Demographic-adjusted discrimination was modest and specificity-oriented. |
| Primary cluster model | Calibration and error | Brier score = 0.218; calibration intercept = -0.000; calibration slope = 1.000; Hosmer-Lemeshow χ² = 14.640, p = 0.067 | No clear calibration departure was detected, but external validation is needed. |
| Sensitivity: HAMD excluded from clustering | K = 3 solution and demographic-adjusted high-risk association | Cluster sizes = 135/26/126; SI rates = 39.3%/65.4%/59.5%; high-risk OR = 2.669 (95% CI: 1.598-4.458), p < 0.001 | The broad cluster-SI association persisted when HAMD was removed from clustering. |
| Sensitivity: HAMD added to association model | Primary high-risk cluster and HAMD total score | High-risk cluster OR = 0.842 (95% CI: 0.411-1.725), p = 0.639; HAMD total score OR = 1.679 (95% CI: 1.484-1.900), p < 0.001 | The cluster association was attenuated after controlling for depressive severity. |

Abbreviations: AUC, area under the receiver operating characteristic curve; HAMD, Hamilton Depression Rating Scale; NPV, negative predictive value; PPV, positive predictive value; SI, suicidal ideation; WSS, within-cluster sum of squares.
